# Hazardous Alcohol Consumption Is a Major Factor in Male Premature Mortality in a Typical Russian City: Prospective Cohort Study 2003–2009

**DOI:** 10.1371/journal.pone.0030274

**Published:** 2012-02-08

**Authors:** Susannah Tomkins, Tim Collier, Alexey Oralov, Lyudmila Saburova, Martin McKee, Vladimir Shkolnikov, Nikolay Kiryanov, David A. Leon

**Affiliations:** 1 London School of Hygiene and Tropical Medicine, London, United Kingdom; 2 Izhevsk State Technical University, Izhevsk, Russia; 3 Social Technologies Institute, Izhevsk, Russia; 4 Max Planck Institute for Demographic Research, Rostock, Germany and New Economic School, Moscow, Russia; 5 Izhevsk Medical Academy, Izhevsk, Russia; Lerner Research Institute, Cleveland Clinic, United States of America

## Abstract

**Introduction:**

Russia has experienced massive fluctuations in mortality at working ages over the past three decades. Routine data analyses suggest that these are largely driven by fluctuations in heavy alcohol drinking. However, individual-level evidence supporting alcohol having a major role in Russian mortality comes from only two case-control studies, which could be subject to serious biases due to their design.

**Methods and Findings:**

A prospective study of mortality (2003–9) of 2000 men aged 25–54 years at recruitment was conducted in the city of Izhevsk, Russia. This cohort was free from key limitations inherent in the design of the two earlier case-control studies. Cox proportional hazards regression was used to estimate hazard ratios of all-cause mortality by alcohol drinking type as reported by a proxy informant. Hazardous drinkers were defined as those who either drank non-beverage alcohols or were reported to regularly have hangovers or other behaviours related to heavy drinking episodes.

Over the follow-up period 113 men died. Compared to non-hazardous drinkers and abstainers, men who drank hazardously had appreciably higher mortality (HR = 3.4, 95% CI 2.2, 5.1) adjusted for age, smoking and education. The population attributable risk percent (PAR%) for hazardous drinking was 26% (95% CI 14,37). However, larger effects were seen in the first two years of follow-up, with a HR of 4.6 (2.5, 8.2) and a corresponding PAR% of 37% (17, 51).

**Interpretation:**

This prospective cohort study strengthens the evidence that hazardous alcohol consumption has been a major determinant of mortality among working age men in a typical Russian city. As such the similar findings of the previous case-control studies cannot be explained as artefacts of limitations of their design. As Russia struggles to raise life expectancy, which even in 2009 was only 62 years among men, control of hazardous drinking must remain a top public health priority.

## Introduction

Russia has experienced massive fluctuations in mortality at working ages over the past three decades. In 2009, despite recent increases, it still had exceptionally low life expectancy at birth for an industrialised country: 62.8 years for males and 74.7 years for females. [1] The mortality fluctuations have been due to a wide range of causes including circulatory, respiratory and digestive diseases as well as those directly related to alcohol such as acute alcohol poisoning. Analysis of these routinely collected mortality data alongside information about patterns and levels of alcohol consumption has led to the conclusion that these fluctuations have been largely, if not entirely, driven by parallel fluctuations in heavy alcohol drinking among people of working-age. [2]

Direct evidence from epidemiological studies for alcohol playing a major role in mortality at working ages in Russia has been limited, as few individual-level studies of this issue have been conducted. The most persuasive evidence to date comes from two case-control studies. In 2007 the Izhevsk Family Study estimated that 43% of deaths among men aged 25–54 years could be attributed to hazardous alcohol drinking [3]; in 2009, a study of a larger number of deaths at working ages in Barnaul, Siberia, concluded that half of all deaths could be attributed to alcohol. [4] Of necessity both of these case-control studies obtained information on alcohol consumption of subjects from proxy informants (mainly family members).

In this context, the case-control design chosen for the initial study had the advantage of being able to determine alcohol consumption in the period immediately preceding death, and hence be sensitive to short-term effects of consumption on mortality. [5] However, studies with this design may suffer from potentially important biases that are difficult to exclude. Firstly, proxy reports of alcohol consumption among cases may be influenced by the fact of death in a way that differs from any over or under-estimation of drinking by proxy informants of live controls. Secondly, selection bias may be introduced because the drinking behaviour of the control series may not be representative of that in the population from which the case series comes. Both biases could result in either under or overestimation of the strength of effect.

It is therefore necessary to complement the earlier case-control study with a cohort study. This has the advantage of avoiding the two very specific problems of case-control studies outlined above. The disadvantage is that unless it is very large, it may be relatively insensitive to short-term effects of alcohol consumption occurring in the first year or two or follow-up. [5] Moreover, depending upon the method of initial recruitment they may tend to differentially exclude heavy drinkers. [6]

In this paper we report the results of a prospective mortality follow-up of working age men from the City of Izhevsk, Russia. The objective of this study was to establish whether using a design without these limitations yielded similar results to those already reported by the two earlier case-control studies.

## Methods

A population-based cohort was constructed of 2000 men who lived in the city of Izhevsk, Russia who were aged 25–54 years at recruitment. Izhevsk has a typical demographic profile for a medium sized-city in Russia. [3] The men were an age-stratified random sample from a register of Izhevsk residents. 1750 men had been originally identified as age-matched live controls for the Izhevsk Family Study (IFS), a case-control study of premature mortality conducted 2003–5. [3] These 1750 live men formed the majority of the cohort, which was supplemented by a further 250 live subjects recruited between November 2006 and January 2007 using an identical approach. At recruitment to study, of the 2000 men, 15.4% were aged 25–34 years, 25.3% 35–44 years and 59.3% 45–54 years.

Information about alcohol drinking and other characteristics was obtained by trained interviewers from proxy informants who lived with the men, the majority (85%) of whom were their wives, partners or girlfriends. In addition we also interviewed the men themselves, although these data were not used in the analyses reported here. This was because we wished to make direct comparisons with the results of the earlier IFS case-control study which of necessity had to use proxy data because the cases were deceased. It should be noted that information about behaviours and drinking habits from proxies in this prospective study was collected prior to the death of the man, and as such could not be biased by the death per se – one of the main weaknesses of the case-control design.

We used the same criteria as previously employed in the case-control study to define types of alcohol drinker. Problem drinking was defined as during the past year having an average of twice-weekly or more occurrences of excessive drunkenness, hangover or going to sleep at night clothed because of being drunk, or one or more episodes of *zapoi*. This is a Russian word used to describe a period of two or more days of continuous drunkenness when the person is withdrawn from normal life. As previously described [5], these questions were specifically designed for use with proxy informants as they asked about behaviours that would be obvious and easily observed by people living with the subject.

One further distinctive aspect of Russian drinking measured was whether the man had consumed non-beverage alcohols at any point in the preceding year. These are manufactured ethanol-based liquids not intended for drinking such as medicinal tinctures that generally do not contain other toxic substances. [7] They have been widely available throughout Russia and per unit of ethanol are a cheaper source of ethanol than any standard beverage. [8] Based on this information, four categories describing drinking type were defined: abstained in previous year; non-problematic drinker of beverages (beer, wine or spirit); problematic drinker of beverages; drinker of non-beverage alcohol. A number of men could not be classified according to their drinking behaviour because the proxy informant did not provide one or more responses to the constituent questions. Education was classified in three categories: incomplete secondary or less; secondary, specialised or vocational; higher, complete or incomplete. Smoking status was classified in three categories: never; ex-smoker; current smoker.

Most deaths were identified by matching names and dates of birth against monthly lists of deaths in the city and surrounding districts produced by the Izhevsk registry office (ZAGS). This was possible for the entire follow-up period with the exception of 2006. In addition, from the end of 2007 to early 2010, we attempted to recontact cohort members as part of a follow-up study. Some of the deaths of study members were notified to the study team by family members or neighbours. Finally, in June 2010 we attempted to recontact all subjects whose vital status at the end of the follow-up period (31 March 2009) remained unknown. This was done by phone or visiting addresses. Using these methods, the vital status of 1956 men was ascertained, with no follow-up information available on only 44 men. In the main analyses these men were assumed to be alive at the end of follow-up. Cause of death was only available for the deaths ascertained through linkage to ZAGS (72/113). For other deaths, only information on date and fact of death was obtained. For this reason we had to restrict our analyses to mortality from all causes combined.

For the majority of deaths (85/113), exact date of death was known, while for a minority (19/113) day, or day and month of death were not known or date was not known at all (9/113). If only day of death was missing, it was imputed to be the middle of the month. If day and month were missing, these were imputed to be the middle of the known year (30 June) or the mid-point between the date of last contact of the man in that year and the end of the year.

### Statistical analysis

The association between type of alcohol drinker, vital status, education, smoking status and age was investigated using chi-square tests. Cohort members were defined as entering risk on the date of their proxy interview and ceased to contribute to risk on their date of death or at the end of follow-up (31 March 2009). A Kaplan-Meier plot was used to visualise survival rates by alcohol consumption category over the follow-up period. Mortality rates per person-year were determined. Cox regression analysis was used to obtain mortality hazard ratios both unadjusted and adjusted for age at entry to the study, education and smoking status using the moderate drinkers as the reference group. Evidence for changes in the effect of alcohol on mortality over the follow-up period (first two years versus subsequent follow-up) was sought *a priori*, as we have previously suggested that some of the effects of hazardous drinking on mortality observed in the case-control study may be relatively acute. [5] A likelihood ratio test was used to test whether the hazard ratios in these two periods differed. Population attributable risk percentages for hazardous and non-beverage drinking were calculated for the whole follow-up time and for the first two years of follow-up using the method proposed by Greenland and Drescher. [9] A number of sensitivity analyses were carried out to investigate potential sources of bias. For those men with whom no contact was made following entry to study we censored follow-up at 1 week and at the median follow-up time for the cohort. Men who were not known to be dead were censored at the date of their last live contact. Finally, we repeated the analysis restricting deaths to those identified via ZAGS and excluding deaths reported by any other informants.

Analyses were conducted using STATA, version 11.

### Ethics statement

The Izhevsk Family Study was approved by the Ethics Committees of the London School of Hygiene and Tropical Medicine and the Izhevsk State Medical Academy. Verbal informed consent was obtained from all subjects. This was obtained in preference to written consent due to awareness of local cultural issues regarding fear of signing official documents, and concerns regarding how this would impact respondent participation. Verbal informed consent was recorded by interviewers on the cover page of the questionnaire before proceeding and this was entered into the database. This procedure was approved by the Ethics Committee of the London School of Hygiene and Tropical Medicine and the Izhevsk State Medical Academy.

## Results

The mean duration of follow-up was 3.99 years (range 0.02 to 5.31). Of the 2000 men 113 were found to have died in the study period. The mortality rate of the cohort remained approximately constant throughout the study period with overall risk of death 1.39 per 100 person years (95% CI 1.15, 1.68), As shown in Table 1 the age-specific mortality rates of the cohort were similar to or higher than that of the City of Izhevsk in 2008.

**Table 1:** Age-specific mortality rates (95% confidence intervals) in the cohort (2003–9) and the City of Izhevsk (2008).

Age group	Number ofmen at baseline	Number of deaths	Rate per 100 person years (95% CI)		City of Izhevsk male mortality rate in 2008 (per 100 population)
30–39	334	17	1.27	(0.79, 2.05)	0.59
40–49	827	36	1.08	(0.78, 1.50)	1.05
50–59	695	54	1.96	(1.50, 2.56)	2.19

Note: the small number of person years and deaths in the cohort occurring in the age-group 25–29 are excluded in order to make direct comparisons with the routinely published data for Izhevsk.

**Figure 1:**
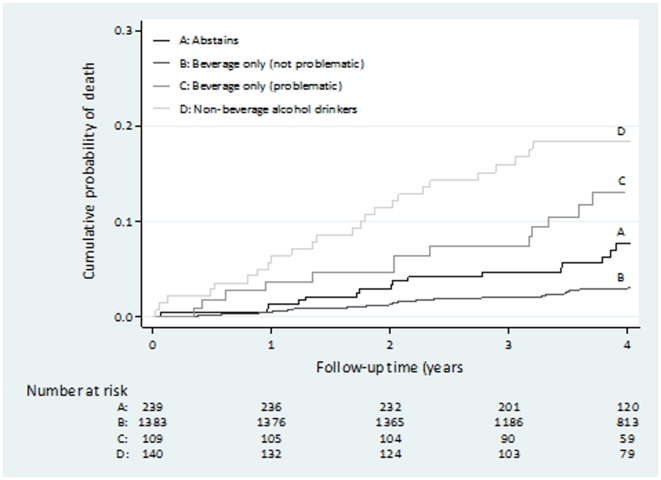
Kaplan-Meier plot of cumulative probability of death over four years follow-up by proxy reported type of alcohol drinking.

When examined by drinking behaviour, the highest mortality risk was observed in the two groups with the most hazardous drinking behaviours (beverage only (problematic) 12.8%; non-beverage drinker 18.6%). Figure 1 shows the cumulative probability of death for each type of alcohol drinker throughout the follow-up period in this prospective study. The probability was higher throughout the follow-up period in the non-beverage drinking category and lowest in the beverage non-problem drinking category. Type of alcohol drinking was associated with both smoking and education (data not shown). Hazardous drinkers were most likely to be current smokers and also be in the lowest categories of educational level (p<0.001). Those who were older at entry to the study were also more likely to be hazardous drinkers (p = 0.01).

Table 2 shows the mortality rates in each alcohol category, and the hazard ratios adjusted for age and then additionally for smoking and education. A strong association of pattern of drinking with mortality was observed, with mortality rates progressively higher in each more extreme alcohol category, and the highest hazard ratio observed for non-beverage drinking. As expected, a high mortality rate was observed for abstainers, consistent with this group including a proportion of former drinkers who gave up drinking due to ill health. Expected associations were seen for smoking and education. Adjustment for smoking and education resulted in a slight attenuation of the hazard ratios, although a strong association was still seen for type of drinking. Pattern of drinking was missing for 129 men, 9 of whom died during follow-up. Relative to beverage-only (non-problematic) drinkers, this group had a mortality hazard ratio of 2.18 (1.07, 4.45).

**Table 2:** Mortality rates and hazard ratios (95% confidence intervals) by proxy reported type of alcohol drinking.

	Number of men at baseline	Number of deaths	Rate(per 100 person years)	Age-adjustedHR (95% CI)	Adjusted^a^HR (95% CI)
**Type of alcohol drinker**					
Abstains	239	17	1.82	2.07 (1.19, 3.62)	2.07 (1.18, 3.62)
Beverage only (not problematic)	1383	48	0.85	1.00 [ref]	1.00 [ref]
Beverage only (problematic)	109	13	3.09	3.52 (1.91, 6.51)	2.91 (1.56, 5.42)
Non-beverage alcohol drinker	140	26	5.04	5.71 (3.54, 9.22)	4.80 (2.93, 7.87)
				p<0.0001^b^	p<0.0001^b^
**Smoking Status**					
Never	403	10	0.61	1.00 [ref]	1.00 [ref]
Ex	240	8	0.83	1.31 (0.52, 3.31)	1.12 (0.44, 2.84)
Current	1228	86	1.76	2.86 (1.49, 5.51)	2.00 (1.02, 3.92)
				p<0.001^b^	p = 0.037^b^
**Education Status**					
Incomplete secondary/lower	100	7	1.71	2.38 (0.92, 6.19)	1.20 (0.45, 3.19)
Secondary	1335	85	1.60	2.43 (1.30, 4.56)	1.72 (0.91, 3.25)
Higher	420	11	0.64	1.00 [ref]	1.00 [ref]
Unknown	16	1	1.71	2.51 (0.32, 19.51)	1.41 (0.18, 11.07)
				p = 0.02^b^	p = 0.29^b^

a adjusted for age, education and smoking status.

b p-value from likelihood ratio test.

Table 3 shows the adjusted effect estimates separately for the first two years and subsequent period of follow-up. There was some suggestion that the association of alcohol with mortality was stronger in the initial two years of follow-up compared to the remaining period. However, a formal test of interaction suggested this difference between hazard ratios could be due to chance.

**Table 3:** Adjusted^a^ mortality hazard ratios (95% confidence intervals) by proxy reported type of alcohol drinking and follow-up period.

Type of alcohol drinker	Follow-up period	
	0–2 yrs	2+ yrs
Abstains	2.19 (0.91, 5.26)	2.00 (0.98, 4.12)
Beverage only (not problematic)	1.00 [ref].	1.00 [ref]
Beverage only (problematic)	2.90 (1.07, 7.85)	2.93 (1.33, 6.44)
Non-beverage alcohol drinker	7.41 (3.73, 14.70)	3.09 (1.49, 6.41)
		p = 0.32^b^

a adjusted for age, education and smoking status.

b p-value from interaction test.

A summary measure of the association of hazardous drinking on mortality may be obtained by calculating the mortality hazard ratio for the combined category of problem beverage and non-beverage drinkers versus the combined category of non-problem beverage drinkers plus abstainers: giving a hazard ratio of 3.4 (2.2, 5.1), adjusted for age, smoking and education. The equivalent hazard ratio for the first two years of follow-up was 4.6 (2.5, 8.2). This binary contrast of hazardous versus non-hazardous drinkers and abstainers resulted in a population attributable risk of 26.3% (14.4, 36.6). For the first two years of follow-up it was 36.5% (17.2, 51.4).

A series of sensitivity analyses were conducted to determine the potential impact of the uncertainty concerning the vital status of a small proportion of study subjects. For the 44 men for whom no contact was made following entry to study, imputing median follow-up time, and alternatively imputing 1 week follow-up time yielded very similar results to the overall findings. In total, 127 men who were not known to be dead had a last live contact prior to the end of follow-up. Censoring these men at the date of their last live contact had a negligible effect on our results. A sensitivity analysis restricting deaths to those identified via ZAGS and excluding deaths reported by any other informants also made very little difference to the overall results.

## Discussion

The strong association of pattern of drinking with mortality in this prospective follow-up study is very similar to that seen in the original Izhevsk case-control study, although the effects in this study are slightly smaller. In the case-control study, the age, smoking and education-adjusted mortality odds ratios obtained comparing groups to beverage (non-problem) drinkers were 1.25 (0.98–1.60) for abstainers, 2.93 (2.22–3.88) for beverage problem drinkers, and 8.64 (6.90–10.8) for non-beverage drinkers. However, the larger effect sizes seen in the first two years of follow-up in the current study are much more similar to those from the case-control study. The population attributable risk percent associated with hazardous drinking in the cohort study was lower than from the case-control study, where it was estimated to be 43%. However, if based on the effects in the first two years of follow-up, the PAR% is again close to that from the case-control study.

In this study the men who were classed as abstainers had twice the mortality of those who were non-problematic drinkers. This finding is consistent with other data from Russia [10] and from many studies elsewhere. [11] The main explanation for this is that people who do not drink at any one point in time are a mixture of life-time abstainers and former drinkers an important proportion of whom stopped drinking because of ill health. It is these “sick-quitters” who drive the raised mortality of this group, particularly in Russia where there are very few life-time abstainers.

One of the very striking aspects of the link between mortality and alcohol drinking in Russia as apparent from routine data, is that mortality from a wide range of causes rise and fall very sharply in response to changes in underlying patterns of alcohol consumption. [12] This is most obvious for causes directly linked to alcohol, but is also seen for other causes, for example for circulatory disease mortality. [13] This suggests that an important part of the burden of alcohol-related mortality in Russia is the result of acute effects of recent episodes of heavy and hazardous drinking.

On this basis, it is to be expected that the strength of association between drinking type and mortality in the first period of follow-up would be larger than for subsequent follow-up periods. Moreover, other things being equal, the cohort study estimates for the first couple of years of follow-up should effectively be estimating the same sort of effect as estimated by the case-control study. This is indeed what we have found (Table 3). Thus, although formally the difference in effect size by period of follow-up could be due to chance, finding this difference is what we expected.

Some methodological aspects of the study deserve comment. The fact that the mortality rates in the cohort are similar, or even slightly higher, than those for the city of Izhevsk confirms that our recruitment strategy was not subject to a “healthy participant” effect. This result also suggests that although our method of ascertainment of deaths over the follow-up period was not ideal, and some cohort members may have in fact died when we thought that they were alive, the scale of this problem is likely to have been minimal. This conclusion was supported by the results of our sensitivity analyses.

The number of deaths included in the analysis was relatively small, resulting in estimates of effect with wide confidence intervals. Despite this, as already noted the point estimates for the first two years of follow-up were very similar to those from the much larger case-control study. From another perspective, it may be argued that this similarity suggests that the Izhevsk case-control study did not suffer from major recall and selection bias. Crucially, in the prospective study, proxy reports of alcohol drinking were obtained prior to death. Either way, the similarity in findings using different designs strengthens the validity of the conclusion that hazardous alcohol drinking as measured in these studies is an important causal factor in explaining working age male mortality in this typical urban Russian population.

Putting the study in a broader context, the follow-up period of the study was from late 2003 to the March 2009. During this time Russian male life expectancy was initially stagnant and then from 2005/6 began to increase. [14] Over this same period, although life expectancy at birth was lower in Udmurtia than in Russia as a whole (Udmurtia is the region of which Izhevsk is the capital), similar trends were seen. Some of this effect appears to be due to the effect of federal measures to more tightly control the manufacture and use of ethanol, particularly in non-beverage alcohols. [2], [8], [15] However, it is also likely that this was accompanied by a reduction in prevalence of hazardous drinking of spirits in general, whether from vodka or non-beverage sources. Despite, these improvements, male life expectancy remains alarmingly low for an industrialised country. There are indications that this is now being considered an important political issue in Russia. These results, underline the importance of continuing to advance policies that will further reduce the level of hazardous drinking in Russia as a central aspect of any realistic strategy to increase life expectancy.
